# Understanding human papillomavirus vaccine response and efficacy in people living with HIV: A systematic mixed studies review and meta-analysis

**DOI:** 10.1371/journal.pgph.0003931

**Published:** 2024-12-20

**Authors:** Alvine M. Akumbom, Alanna J. Bergman, Howard Strickler, Chakra Budhathoki, Manka Nkimbeng, Raeven Grant, Nancy R. Reynolds, Kawsar R. Talaat

**Affiliations:** 1 Center for Infectious Disease and Nursing Innovation, Johns Hopkins School of Nursing, Johns Hopkins University, Baltimore, Maryland, United States of America; 2 University of Virginia School of Nursing, Charlottesville, Virginia, United States of America; 3 Department of Epidemiology and Population Health, Albert Einstein College of Medicine, Bronx, New York, United States of America; 4 Division of Health Policy and Management, University of Minnesota School of Public Health, Minneapolis, Minnesota, United States of America; 5 David Geffen School of Medicine at the University of California Los Angeles, Los Angeles, Los Angeles, California, United States of America; 6 Department of International Health, Center for Immunization Research, Johns Hopkins Bloomberg School of Public Health, Baltimore, Maryland, United States of America; Chinese Academy of Medical Sciences and Peking Union Medical College, CHINA

## Abstract

Coinfection with human papillomavirus (HPV) and HIV compounds the risks of developing cervical, anal, and HPV-associated oral neoplasia. Safe prophylactic vaccines are available to prevent HPV infections in people with HIV(PWH). Yet, vaccine efficacy and duration of protection remain questionable. Historically, the efficacy of vaccines has been suboptimal in PWH compared to people without HIV (PWoH).A systematic review of HPV vaccine trials in PWH was conducted using PRISMA guidelines. Outcomes of interest were vaccine efficacy, immunogenicity, and predictors of HPV vaccine efficacy. A secondary outcome was to assess age and sex differences. Efficacy was reviewed as cervical/anal/oral lesions or neoplasia, and incident or persistent HPV infection following vaccination. A random effects meta-analysis was performed comparing geometric mean titer (GMT) in PWH to PWoH. Twenty-eight studies out of 988 were eligible for inclusion in our study, and qualitatively synthesized. Eight of these studies were meta-analyzed. GMT results of HPV16 and HPV18 genotypes were significantly lower in PWH; Hedges’s g -0.434 (95% CI: -0.823, -0.046) and Hedges’s g -0.57 (95% CI: -0.72, -0.43), respectively. The mean difference in GMT for HPV18 between PWH and PWoH was -536.23 (95% CI: -830.66, -241.81); approximately 22 times higher than HPV18 seropositivity cut-offs, assuming milli-Merck Units per milliliter. Risk factors for incident or persistent infections in PWH included: failure to seroconvert after vaccination, baseline CD4+ T-cell count <500 cells/mm3, early age of sexual debut, HIV viral load ≥ 400 copies/mL. There was a trend towards decreased HPV vaccine efficacy in studies that included enrollees with a history of AIDS or AIDS-defining illness.Applying existing evidence of HPV vaccine efficacy on meaningful clinical outcomes in PWH is questionable. This could be influenced by the diversity of eligibility criteria across clinical trials of HPV vaccine efficacy. Precision medicine may offer novel alternatives for evaluating HPV vaccine efficacy in PWH.

## I. Introduction

Human papillomavirus (HPV) is a vaccine-preventable viral infection that leads to cervical, anogenital, and oral neoplasia [1,2]. Although most HPV infections clear spontaneously, HPV remains the most common sexually transmitted infection globally with persistent infection causing premalignant and malignant lesions that disproportionately affect people with HIV(PWH). PWH are highly susceptible to HPV infection, persistence, and progression to neoplasia. The tat and gp120 proteins located on HIV-1 genes distort the epithelial tight junctions in the mucosal epithelium [3]. This eases the entry of HPV and enables development of neoplasia in PWH [3,4]. Consequently, HIV increases the risk of cervical, oral, and anal cancers as well as other diseases caused by HPV.

Generally, most cancers caused by HPVs occur in women. This is primarily due to the global burden of cervical cancers, estimated at 660,000 new cases and 350,000 deaths in the year 2022 [2]. Cervical cancer accounts for 47% of all HPV cancer cases in the United States, while anal cancer constitutes 20% of cases, and oropharyngeal cancer represents 14% of cases [1]. The other HPV-associated cancers (penile, vaginal, vulval), not discussed in this manuscript, constitute the remaining approximately 19% of HPV-associated cancers reported in the United States. Oral cancer is most common in men, while anal cancer is most common in women. The United States estimates 12,500 oral cancer cases in men versus 2,300 oral cancer cases in women, and 2,200 anal cancer cases in men versus 4,700 anal cancers in women, per annum [1].

Globally, women living with HIV (WLWH) have the highest risk of developing cervical cancer. A meta-analysis attributed a six-fold risk of cervical cancer in WLWH compared to women without HIV (WLWoH) [5]. Notably, regional and sex differences also exist in the distribution of the burden of HPV infections and HPV-attributable lesions in PWH. For example, in Southern Africa, 63.8% of women with cervical cancer are also living with HIV [5]. Meanwhile, the prevalence of oncogenic HPV strains in men living with HIV was estimated at 46.9% in China [6], and at 65.3% of men living with HIV who have sex with men in Canada [7]. Safe, prophylactic vaccines are available to protect against significant oncogenic HPV strains.

### Overview of HPV vaccines

There are three prophylactic HPV vaccines licensed for global marketing: the bivalent vaccine (2vHPV vaccine or Cervarix by GlaxoSmithKline) [8], the quadrivalent vaccine (4vHPV vaccine or Gardasil by MERCK & Co., INC.) [9], and the nonavalent vaccine (9vHPV vaccine or Gardasil 9 by Merck & Co., INC.) [9]. The 2vHPV and 4vHPV are first-generation HPV vaccines that prevent infection against two oncogenic types: HPV 16 and 18. The 4vHPV also prevents HPV types 6 and 11 that cause genital warts. The 9vHPV vaccine is a second-generation vaccine that offers protection against oncogenic and nononcogenic types 6, 11, 16, 18, 31, 33, 45, 52 and 58. HPV types 16 and 18 account for about 70% of cervical cancers [1,2,10]. HPV types 31, 33, 45, 52, and 58 cause an additional 12% to 20% of cervical cancer cases [1,11,12]. Only genotypes16 and 18 areincluded in all three licensed HPV vaccines. HPV vaccines are licensed for prophylactic use, not therapeutic interventions. Availability and distribution of these vaccines vary by country. In the United States, for example, only the 9vHPV vaccine is currently recommended and available for use. Alternatively, in South Africa, the 2vHPV is available via public schools, while both the 2vHPV and 4vHPV vaccines are available via the private sector [13,14].

### HPV vaccination recommendation for PWH

Due to the significant protection against high-risk HPV strains from HPV vaccines, the World Health Organization (WHO) recommends vaccination for all adolescents living with HIV as a two-dose to three-dose schedule (0, 1–2, 6 months), irrespective of whether they are on antiretroviral therapy (ART) [15]. Aligning with the focus on cervical cancer prevention globally, the WHO recommends two doses, with a six-month interval for women older than 21, and two to three doses for immunocompromised individuals [15]. Meanwhile, The Centers for Disease Control and Prevention (CDC) recommends vaccination of adults up to 45 years. The recommendations for adults encourages clinicians to engage in shared-decision making with individuals ages 27 through 45 years, to determine their likelihood of benefiting from HPV vaccination [16].

HPV vaccines may be less efficacious in PWH, as decreased efficacy of other vaccines is seen in PWH such as vaccination against influenza and pneumococcal pneumonia [17,18]. Nonetheless, given that the licensed HPV vaccines confer significant protection against high-risk HPV strains, vaccination should be prioritized for PWH.

### Recent reviews

The safety and tolerability of HPV vaccines in PWH have been thoroughly discussed in recent reviews, both qualitatively and quantitatively [19,20]. These referenced reviews were both systematic reviews of randomized clinical trials (RCT). In their meta-analyses, Zizza and colleagues also analyzed aggregate immunogenicity outcomes on the mean difference in geometric mean titers (GMT) comparing HPV vaccines to placebo in PWH, and reported evidence of significantly higher antibody levels among vaccine recipients [20]. Only one efficacy trial was included among the RCTs reviewed. The study reviewed reported efficacy on HPV anal and oral infections, and anal high-grade squamous intraepithelial lesions [20,21]. Cervical intraepithelial neoplasia (CIN) was not measured as an outcome in the RCTs for PWH despite the high burden of cervical cancer in WLWH.

### Importance of this review

RCTs for HPV vaccine clinical trials rarely follow participants long enough to evaluate efficacy against neoplasia endpoints given the lengthy duration from infection to development of malignancy. Hence, traditional RCTs often lack efficacy data on persistent HPV infections and neoplasia outcomes in PWH. The licensed HPV vaccines were determined to be safe, immunogenic, and efficacious against neoplasia in the general population [22,23]. Thus, it is now unethical to randomize PWH to vaccine groups compared to placebo. As such, a review that includes HPV vaccine clinical trials without a placebo arm is essential to understand the long-term impact of HPV vaccination on viral and clinically relevant endpoints in PWH. A recently published review validated evidence on seroconversion and seropositivity in PWH from both RCTs and prospective longitudinal studies [24]. With the increased risk of cervical and anal intraepithelial neoplasia among PWH, and the duration of progression to premalignant or malignant tumors, it is essential to assess the long-term benefits of HPV vaccines in PWH.

### Objectives of our review

The primary objectives of this review were: (1) to evaluate the immunogenicity, clinical efficacy, and duration of protection of HPV vaccines in PWH, and (2) to investigate HPV vaccine response by HIV serostatus. The secondary objective of our study was to assess the differences in HPV vaccine response by age and sex.

## II. Methods

### Information sources and search strategy

This review was guided by the Preferred Reporting Items for Systematic Reviews and Meta-Analyses (PRISMA) [25,26]. A completed PRISMA checklist is available as (S1 Checklist). The authors collaborated with a library informationist to develop search strategies for three databases: Embase, PubMed, and SCOPUS. The search strategy for Embase is provided for reference as a (S1 Table). We also hand-searched the clinicaltrials.gov website for online records of trial results on HPV vaccination in PWH. Studies were imported into Covidence for deduplication and screening [27]. The initial retrieval of studies from these databases was done on 17^th^ September 2020, and an updated search was conducted on 24^th^ September 2023.

### Inclusion and exclusion criteria

HPV vaccine clinical trials were included if their use was prophylactic, the study included PWH, the study met the National Institutes of Health (NIH) definition of a clinical trial [28], and if the study measured efficacy or immunogenicity as outcomes. NIH’s definition of clinical trials includes both randomized controlled trials and open-label trials. Animal trials were excluded as well as literature reviews, conference abstracts, and non-English publications. We also excluded publications that reported safety and tolerability information only (without outcome data), as safety and tolerability of HPV vaccines in PWH have already been thoroughly reviewed. All eligible studies, regardless of publication date, were considered for inclusion. This review does not have a registered protocol.

### Study selection and risk of bias

Three reviewers (A.M.A., A.J.B., and M.N.) independently reviewed study titles and abstracts, then thoroughly screened each full text article resolving conflicts through discussion and consensus. The reviewers independently extracted data into predeveloped forms for comparison and analysis. Efficacy was reviewed as the presence cervical/anal lesions or neoplasia, oral lesion or neoplasia, and incident or persistent HPV infection following vaccination. The primary immunogenicity endpoints were seroconversion to HPV types 16/18 and GMT for HPV types 16/18 at month seven (or four weeks after the third dose). All reviewers appraised each study using the Cochrane risk of bias tool for RCTs, and the ROBINS-I tool for assessing risk of bias in non-randomized studies of interventions [29,30]. The risk of bias assessment was completed at the study level, and data presented with the aid of a data visualization figure. The risk of bias assessment visualization was generated using the robvis software [31].

### Quantitative data extraction

**Fig 1:**
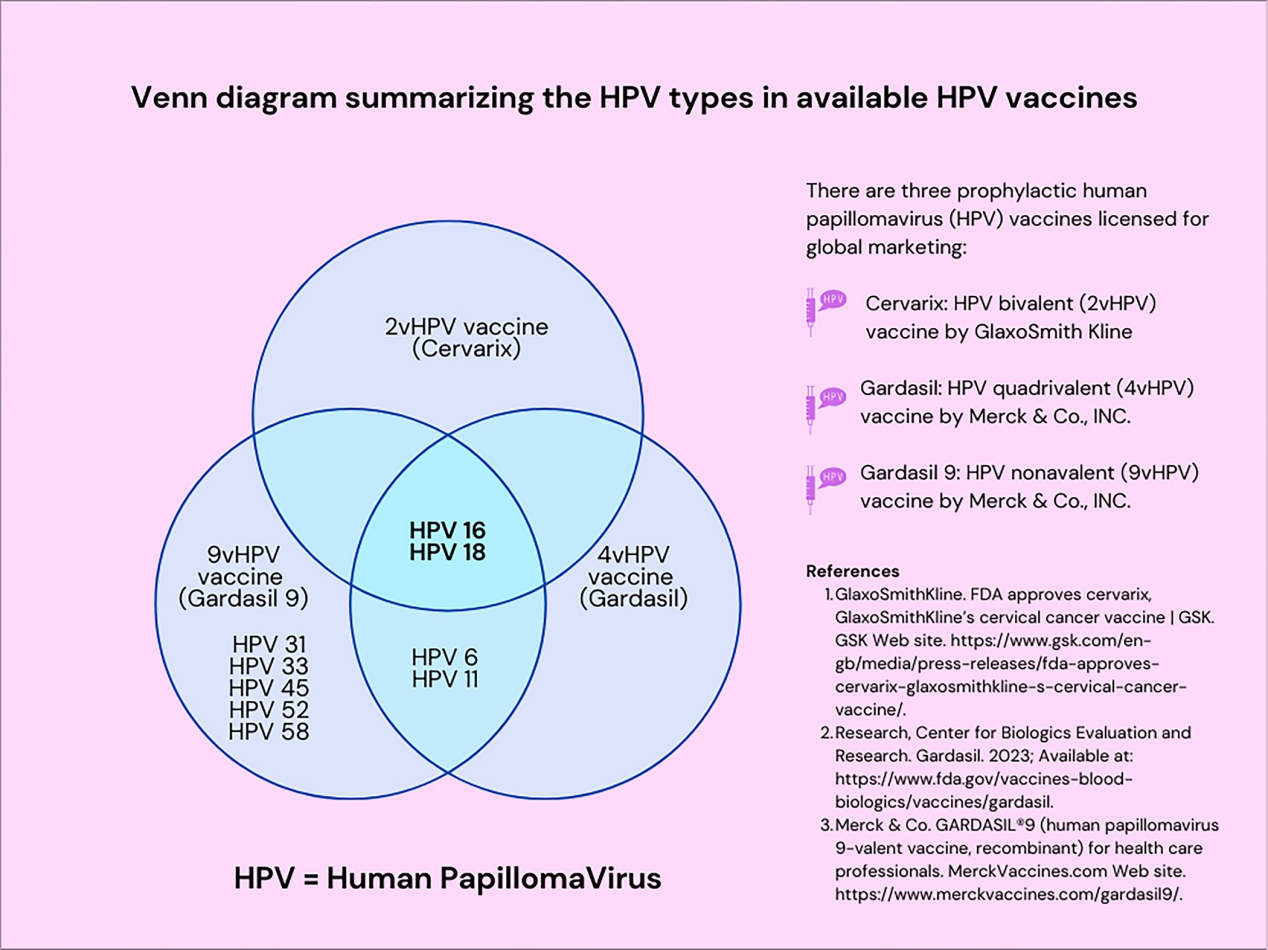


Full text articles were evaluated to extract data points for meta-analysis or quantitative synthesis by A.M.A. and R.G., independently. Some of the variables extracted included sample size, the total number of participants vaccinated, vaccine type, total PWH by sex, and immunogenicity results at month 7 and subsequent time points. Most studies used the pre-licensure definitions for seropositivity cut-off. These serostatus cutoffs for GMT set by MERCK were previously validated using the competitive Luminex Immunoassay (cLIA) and reported in milli-Merck Units per milliliter (mMU/ml) cite. These values are 20, 16, 20, and 24 mMU/ml for HPV 6, 11, 16, and 18, respectively. We further extracted GMT and their confidence intervals for HPV 16 and 18 for both PWH and PWoH. We only extracted for HPV16 and HPV 18 types because, as aforementioned, these are the only HPV types covered in all licensed vaccines. We summarize the latter in Fig 1. GMT were extracted as reported by the studies reviewed. No GMT values were generated for the purposes of this systematic review. Only studies that reported numeric GMT data on HPV vaccine-specific strains were included in the meta-analysis. GMT results were synthesized by assay type. Where possible, quantitative data on efficacy endpoints were also extracted. Due to substantial differences in GMT and efficacy outcome reporting across the studies, not all available data could be meaningfully synthesized quantitatively. The efficacy of HPV vaccines in PWH could not be meta-analyzed because the quantitative efficacy outcome data across the studies reviewed were uniquely reported within each study which hindered a quantitative comparison. However, all studies that met our inclusion criteria were qualitatively synthesized in our review.

### Quantitative data analysis

The quantitative data validated by A.M.A and R.G. were analyzed by A.M.A and the analyses were verified by C.B. The meta-analysis in our review of immunogenicity was limited to vaccinated individuals where we examined the mean difference in the GMT response (titer levels) to the vaccines by HIV serostatus. Immunogenicity results comparing seroconversion (percentages) in vaccinated PWH and placebo recipients have been thoroughly reviewed both qualitatively and quantitatively in recent publications [19,20].

Meta-analysis was conducted using Stata 18 [32]. We pooled estimates using the restricted maximum likelihood random effects model (REML) while assuming unequal variances [33]. A random-effects model assumes some degree of imprecision or variation between the studies included in our meta-analysis. Thus, our choice of a REML takes into account the between study heterogeneity of HPV vaccine clinical trials of PWH which could result from both observable and unobservable variables that influence the immune response in PWH to HPV vaccines. We compared month seven GMT values for PWH to those of PWoH. We evaluated the effect of age on post-vaccination titers and assessed differences in GMT levels between HPV16 and HPV18 genotypes in PWH. A sensitivity analysis was conducted using a cumulative meta-analysis ordered on age and stratified by assay type. GMT results were synthesized by assay type due to potential differences in GMT with use of different assays. Age cohort stratification is important in evaluating HPV vaccine responses due to the general decline in HPV vaccine efficacy in adults compared to children. Publication bias was assessed using funnel plots. Forest plots are used to visually present results as mean differences in GMT and their 95% confidence intervals (95% CI). Heterogeneity statistics [34], I^2^ and H^2^ are also reported in the Forest plots.

## III. Results

### i. General overview of studies reviewed

#### Selected studies

**Fig 2:**
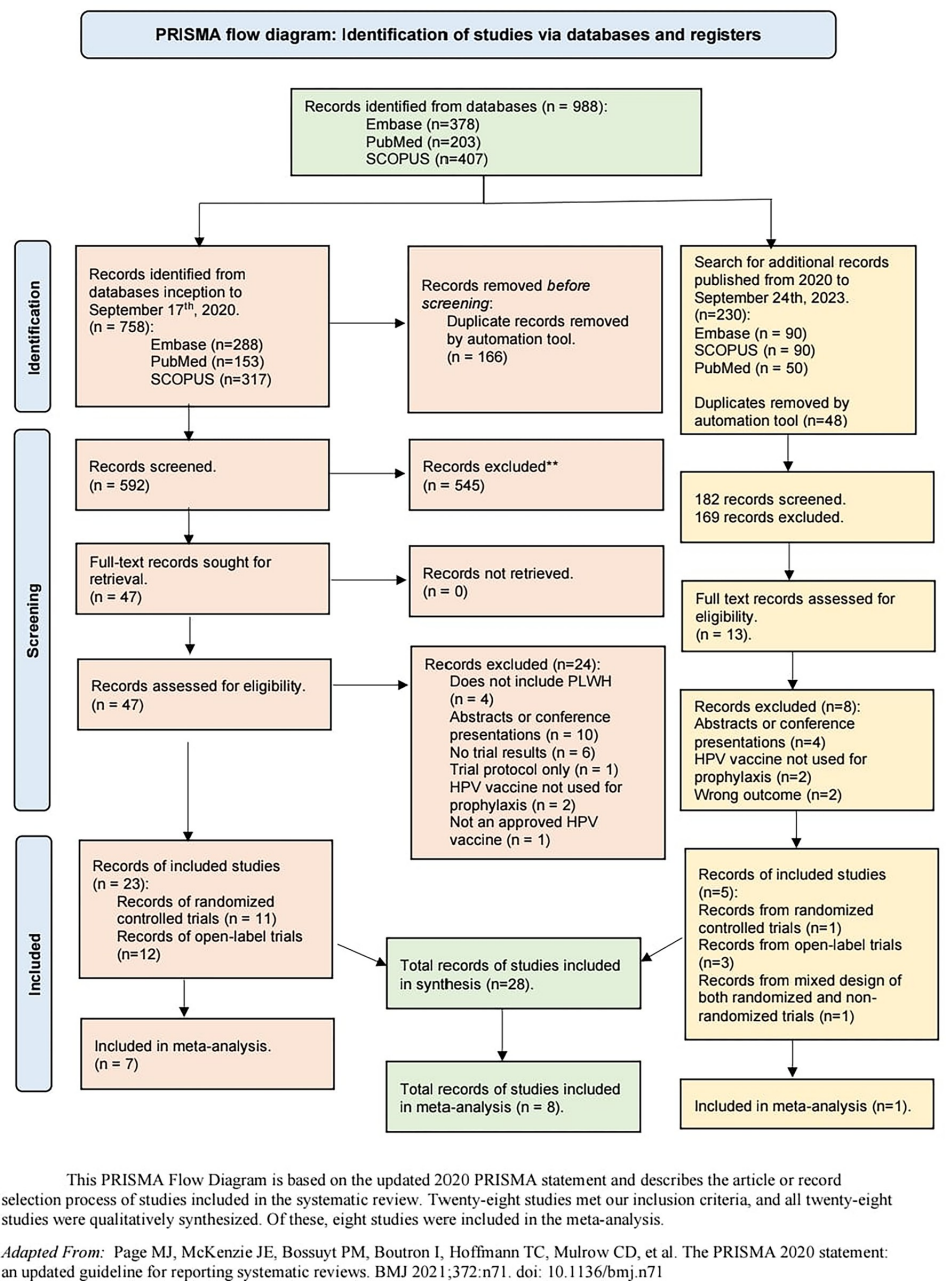
PRISMA flow diagram: Identification of studies via databases and registers.

A total of 988 records were retrieved: 758 from our initial search on September 17^th^ 2020, and 230 from our updated search on September 24^th^ 2023. Sixty studies were determined to be eligible for full text review. Of these 60 studies, 28 studies were included in this review **(Fig 2**). Fig 2, the PRISMA flow diagram depicts our selection process, with reasons for excluding studies. Due to the limited availability of comparable outcome data relevant to our review, seven studies were included in the meta-analysis comparing month seven GMT in PWH to PWoH. Eight studies were included in the meta-analysis comparing post-vaccination titers for HPV16 and HPV18.

#### Study characteristics

Among the included studies, there were 12 publications resulting from seven RCTs, and 15 publications from non-RCTs published between 2010 and 2022. These clinical trials were conducted across thirteen different countries (North America (Canda, Mexico, United States of America), South America (Brazil), Africa (Kenya, South Africa), Europe (Belgium, Denmark, Estonia, Italy, Spain), South Asia (India), and Southeast Asia (Thailand)). Another study which analyzed individual-level data from three clinical trials (clinical trials already included in this review) was also added in our qualitative synthesis [35]. There was unanimous agreement among screening team members for the added value of including this study in our review.

#### Study interventions

The interventions included intramuscular (IM) administration of three doses of 0.5 ml of HPV vaccine (2vHPV or 4vHPV or 9vHPV vaccine), except one study that compared three to four doses of 4vHPV vaccine in young children. The maximum follow-up time was five years [36]. A summary of the general characteristics of studies reviewed is presented in **Table 1** and immunogenicity information is summarized in **S2** Table.

**Table 1:** Summary of characteristics of studies reviewed.

First author, year	Country	Sample size	Study Design Randomized vs non-randomized trial	Age of study participants	Male vs Female participants	Includes Pediatric population	Includes an HIV negative comparison group	Intervention (vaccine and # of doses)	Immunogenicity outcomes assessed	Efficacy outcomes assessed (Y/N)
**A. Randomized controlled trials**										
**Denny, 2013 [37]**	South Africa	N = 150	RCT	Mean age = 21.6 years (Range: 18 to 25 years).	Female	--	✓	• 2vHPV or placebo (Al[OH]3) • 3 doses at M 0/1/6	• Seropositivity rates at M 0/2/6/12 • GMT at M 0/2/6/12	N
**Faust, 2016 [38]**	Denmark	N = 91	RCT	Mean age = 46 years	Male and female	--	--	• 4vHPV or 2vHPV • 3 doses at D0/45/180	• Seroconversion rates at M12 • Antibody response at M12	N
**Folschweiller,** **2020 [39]**	• Brazil • Estonia • India • Thailand	N = 546	RCT	The mean age of WLWH = 20 years. Mean age of WLWoH = 19 years.	Female	✓	✓	• 4vHPV or 2vHPV • 3 doses at M0/1.5/6	• Seroconversion percent at M0/1.5/2.5/7/12/18/24 • Immunogenicity of 2vHPV vs 4vHPV for women with and without HIV	N
**Hidalgo-Tenorio, 2017 [40]**	Spain	N = 129	RCT	Mean age = 37.9 years	Male	--	--	• 4vHPV or placebo • 3 doses at M0/2/6	• Seroconversion rate at M7	N
**Hidalgo-Tenorio, 2021 [41]**	Spain	N = 129	RCT	Mean age = 38.8 years (SD: 10.4)	Male	--	--	• 4vHPV or placebo • 3 doses at M0/2/6	• Seropositivity to any vaccine-type HPV at M7/12/24/36/48	Incident HPV, HSIL at M 12/24/36/48
**Levin, 2010 [42]**	N/A	N = 126	RCT	Mean age = 10 years	Male and female	✓	--	• 4vHPV or placebo • 3 doses at M0/2/6	• Seroconversion rates at M7 • GMT at M7	N
**Munk-Madsen, 2018 [43]**	Denmark	N = 30	RCT	Median ages: 4vHPV = 45 years (age range of 34.0 to 60.0). 2vHPV = 46 years (age range of 38.0, 53.0).	Male and female	--	--	• 4vHPV or 2vHPV • 3 doses at M0/1.5/6	• Seroconversion rate at M7 and M12 • GMT at M7 and M12 • Comparison of reactive, HPV L1 specific CD4 T cells pre and post vaccination.	N
**Toft, 2014a [44]**	Denmark	N = 92	RCT	Median age of 2vHPV group = 47 years (IQR: 38.6–54.2). Median age of 4vHPV = 44.5 years (IQR: 38.2–51.9)].	Male and female	--	--	• 4vHPV or 2vHPV M0/1.5/6	• Seropositivity at M0/7/12 GMT at M0/7/12	Y- anogenital HPV DNA at M7 compared to baseline
**Toft, 2014b [45]**	Denmark	N = 91	RCT	Median age of 2vHPV = 47 years (IQR:38.6–54.2). Median age of 4vHPV = 44.5 years (IQR: 38.2–51.9)]	Male and female	--	--	• 4vHPV or 2vHPV 3 doses at M0/1.5/6	Cross-reactive neutralizing antibodies for HPV 31/33/45 at M0/7/12	Y–incident and persistent anogenital HPV DNA at M7
**Weinberg, 2012 [46]**	United States of America	N = 126	RCT	Median age = 12 years (SD: 1.3; Range: 7 to 12).	Male and female	✓	--	• 4vHPV (immediate treatment) or placebo (deferred treatment) • 4 doses at M0/2/6/24 Immediate vs 3 doses deferred treatment at M24/26/30	Serum and mucosal seropositivity 72 weeks post completion of the 3^rd^ dose	N
**Weinberg, 2018 [36]**	United States of America	N = 74	RCT	Median age = 12 years.	Male and female	✓	--	• 4vHPV • 4 doses at M0/2/6/24 or 3 doses at M24/26/30	• Magnitude and persistence of HPV16/18 post vaccination • IgG, IFNγ and IL2 response at 2-, 3.5-, and 4-5-years post vaccine series completion	N
**Wilkin, 2018 [21]**	• Brazil • United States of America	N = 575	RCT	Median age of 47 years (IQR:40, 52).	Male and female	--	--	• 4vHPV or placebo • 3 doses at M0/2/7	• Seropositivity rates at M7	Y–New persistent HPV infection, persistent oral infection, anal cytology and detection of bHSIL after 52 weeks
**B. Non-randomized/open-label clinical trials (Prospective)**										
**Boey, 2021 [47]**	• Belgium	N = 271	Non-randomized	Median age = 42 years (Range: 18 to 55)	Male and female	--	✓	• 9vHPV • 3 doses at M 0/2/6	• Seroconversion rates at M7 • GMT at M7 • Compared HIV+ to solid organ transplant recipients	N
**Brophy, 2018 [48]**	• Canada	N = 35	Non-randomized	Median age = 11.3 years (Range: 9 to 13)	Female	✓	--	• 4vHPV • 3 doses at M 0/2/6	• Seroconversion rates at M7 • GMT antibody response and M7 and M24	N
**Cespedes, 2018 [49]**	• Brazil • South Africa • United States of America	N = 315	Non-randomized	Median age = 36 years	Female	✓	--	• 4vHPV • 3 doses at M 0/2/6	• Serostatus at week 72 • GMT at M6 and M18 • Compared across CD4 strata (>350, 200–350, <350)	Y–HPV DNA detection in cervix and rectum
**Fontes, 2016 [50]**	Brazil	N = 24	Non-randomized	Mean age = 30.4 years (Range: 18 to 45)	Male	--	✓	• 2vHPV • 3 doses at M0/1/6	• Seroconversion rate at M7 • Compared across CD4 strata (≥500 and <500u/dl)	N
**Giacomet, 2014 [51]**	Italy	N = 92	Non-randomized	Mean age = 20 years (Range: 13 to 27).	Male and female	✓	✓	• 4vHPV • 3 doses at M0/2/6	• Seroconversion rate at M7 • Anti-HPV IgG titers at M 1/3/7/12/18	N
**Kahn, 2013 [52]**	United States of America	N = 99	Non-randomized	Mean age = 21.4 years	Female	✓	--	• 4vHPV • 3 doses at M0/2/6	• Serostatus • GMT • Compared participants with HIVVL >400 to HIVVL<400	N
**Kojic, 2014 [53]**	• Brazil • South Africa • United States of America	N = 319	Non-randomized	Median age = 36 years	Female	✓	--	• 4vHPV • 3 doses at M0/2/6	• Seroconversion rates at M7 • GMT at M7	N
**McClymont, 2019 [54]**	Canada	N = 420	Non-randomized	Median age = 39 years (IQR 34 to 45; range 13 to 66)	Female	✓	--	• 4vHPV • 3 doses at M0/2/6	Not assessed	Y–Detection of vaccine specific HPV types >6 months apart (or at last visit), CIN2+, and genital warts
**McClymont, 2020 [55]**	Canada	N = 284	Non-randomized	Median age = 38 years (IQR: 32 to 44).	Female	✓	--	• 4vHPV • 3 doses at M 0/2/6 `	Not assessed	Y–Detection of persistent oncogenic non-4vHPV. Strains assessed: HPV31/33/35/39/45/51/52/56/58/59/68. 4 years follow-up.
**Money, 2016 [56]**	Canada	N = 372	Non-randomized	Median age = 38 years (IQR: 32–45)	Female	✓	--	• 4vHPV • 3 doses at M0/2/6	• Seroconversion rates at M7 and M24 • GMT at M7 and M24	N
**Moscicki, 2019 [57]**	United States of America	N = 458	Non-randomized	Mean age = 17.7 years (SD: 2.4)	Male and female	✓	✓	• N/A–youth previously received 1–3 doses of Gardasil	• Seropositivity and GMT among previously vaccinated youth in the trial	Y–abnormal cervical cytology and genital warts
**Mugo, 2018 [58]**	Kenya	N = 180	Non-randomized	Median age = 12 years (Range: 9–14)	Male and female	✓	--	• 4vHPV • 3 doses at M0/2/6	• Seroconversion rate at M7 and M12 • GMT at M7 and M12	N
**Palefsky, 2021 [59]**	• United States of America	N = 149	Non-randomized	Median age = 23 years (Range: 18 to 26)	Male	--	✓	• 4vHPV • 3 doses at M 0/2/6	• Seroconversion at M 7 • GMT at M 7/12/18/24 • M 7/24 GMT compared by age (<24, >/ = 24), CD4 (≤500, >500 cells/ mm3), and HIV viral load (</ = 75 or >75 copies/mL).	Incident and persistent perianal 4vHPV strains
**Pinto, 2019 [60]**	• Mexico • United States of America	N = 150	Non-randomized	Median age = 45 years (Range: 22–61)	Male	--	✓	• 4vHPV • 3 doses at M0/2/6	• Systemic and oral seropositivity at M0/7/18 • GMT at M0/7/18	N
**Wilkin, 2010 [61]**	United States of America	N = 112	Non-randomized	Median age = 44 years (IQR: 37–51; Range: 22 to 61)	Male	--	--	• 4vHPV • 3 doses at M0/2/6	• Seroconversion at M7 • GMT at M7	Y–HGAIN or HSIL at M7 and HPV16/18 DNA at M7
**C. Retrospective mixed study (using individual-level data of randomized and non-randomized clinical trials)**										
**Kang, 2022 [35]**	Brazil United States of America South Africa	N = 575	Mixed individual-level data from previously published clinical trials	Median age = 42 years (IQR: 35 to 48)	Male and female	--	--	• M 0/2/6	• Sex differences in antibody titer responses • Antibody titers at M 0/7/18	N

#### Meta-analysis

Eight studies were included in the meta-analysis comparing HPV16 and HPV18 GMT in PWH [37,42,47,48,52,56,58,60]. Seven studies were included in the meta-analysis comparing GMT of PWH to PWoH GMT [37,42,47,48,56,58,60]. Three studies used historical comparable controls of HIV-negative participants [42,56,58]. Mugo et al. (2016) restricted their data on comparative controls to participants from Sub-Saharan Africa (Senegal and Kenya) because the research on PWH was conducted in Kenya [58,62]. This was to control for regional differences that could influence outcomes. For the study by Money et al. (2016), we only extracted data on those <26 years old for the meta-analysis. This was doneto avoid double counting of observations given that their results were stratified by age (15 to 26 years old, and 24 to 45 years old) [56]. There was considerable heterogeneity in GMT reporting across studies comparing PWH to PWoH.

#### Risk of bias within studies

**Fig 3:**
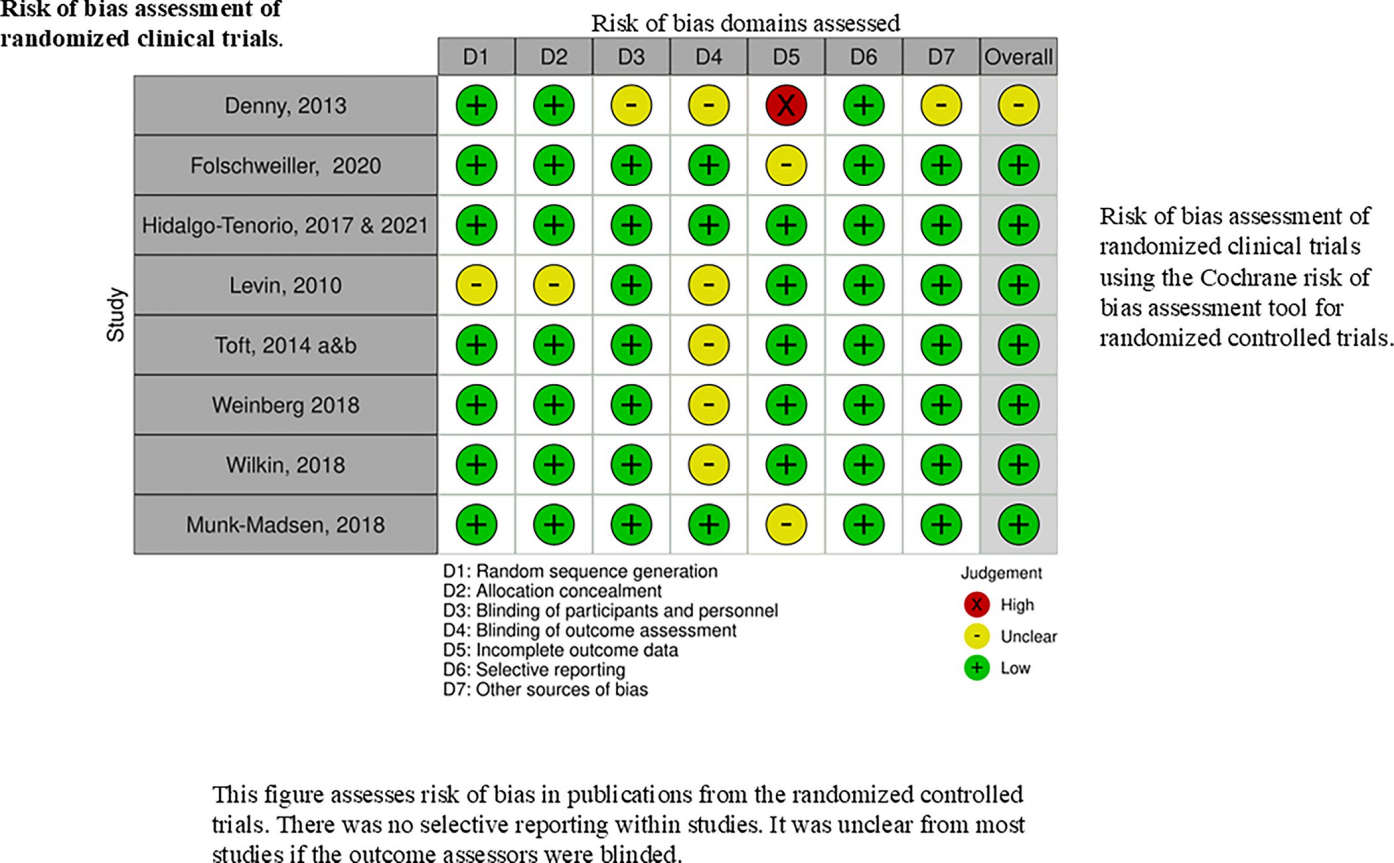


**Fig 4:**
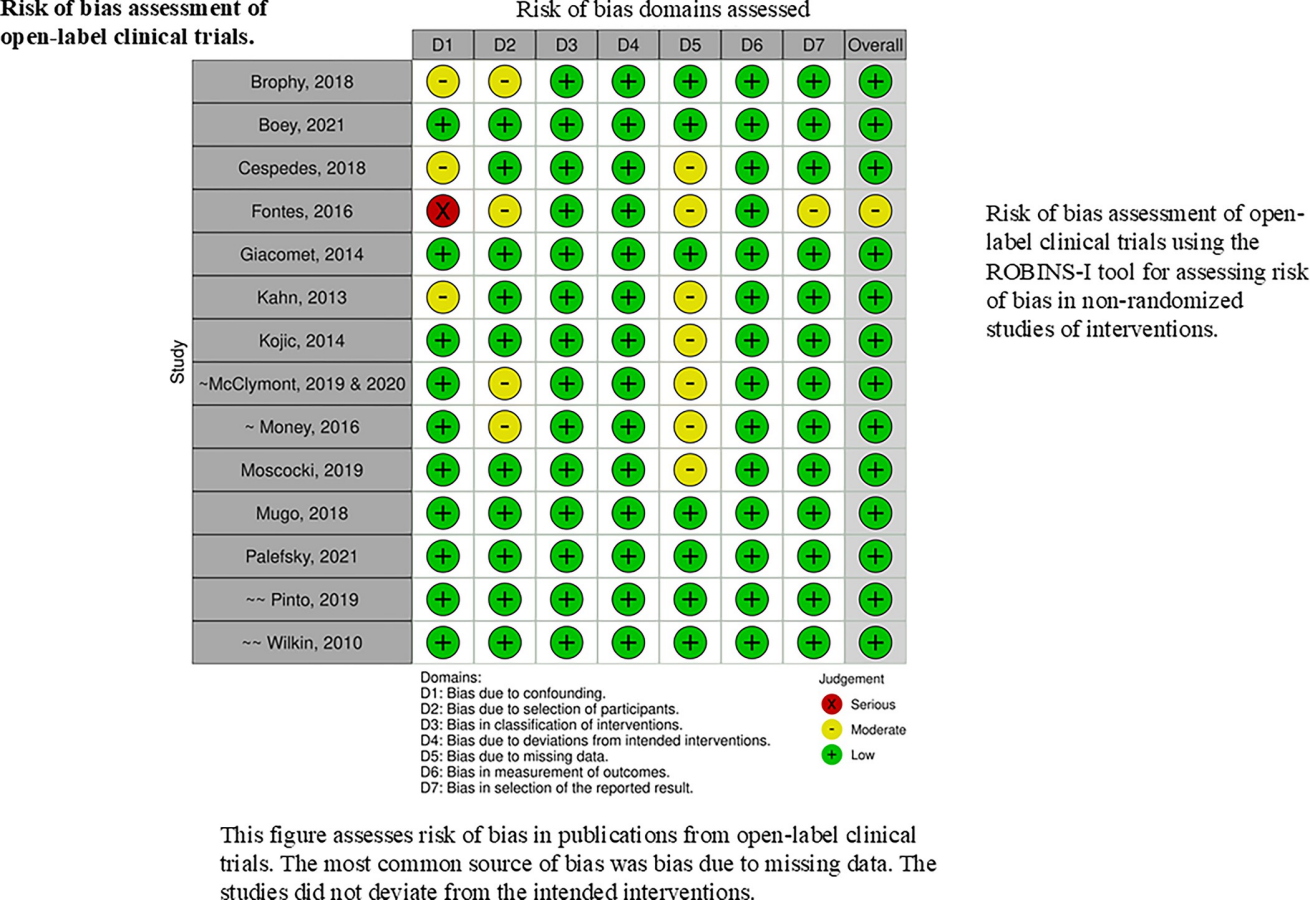


All of the clinical trials that met our inclusion criteria were eligible for inclusion in this review. None of the eligible studies had an overall high risk of bias rating. Thus, none was excluded on the basis of quality. The most common source of bias assessed within the RCTs was the lack of information on whether the outcome assessors were blinded or not. Confounding within studies was reported to be minimized by two common approaches. One was utilizing stringent eligibility criteria, and the other was adjusting for significant confounders in their analysis. The inclusion and exclusion criteria used by some investigators were vague. The most common source of missing data resulted from attrition or lack of outcome data. This review’s risk of bias assessment for included studies is visually presented in **Figs 3 and 4**.

### ii. Data synthesis

#### Immunogenicity

Seroconversion. In general, immunologic response favored PWoH compared to PWH, irrespective of age. However, seroconversion and GMT for 9vHPV vaccine strains were suboptimal in solid organ transplant (SOT) recipients when compared with PWH [47]. Only 46% of SOT recipients seroconverted to HPV45, as opposed to 100% seroconversion in PWH [47]. A myriad of factors could influence the suboptimal HPV vaccine response in the SOT recipients in this study. Suboptimal immunogenicity of HPV vaccines was previously documented in a study of SOT recipients evaluating the immunogenicity of the 4vHPV vaccine [63].

#### Meta-analysis of GMT response in PWH and PWoH

Overall, GMT for both HPV16 and HPV18 genotypes were comparable between PWH and PWoH; Hedge’s g -0.11 (95% CI: -0.83, 0.62) and Hedge’s g -0.22 (95% CI: -0.86, 0.42), respectively. However, after removing solid organ transplant recipients from the group of PWoH, the results significantly favored PWoH with a moderate effect size; Hedges’s g -0.434 (95% CI:-0.823, -0.046) and Hedges’s g -0.57 (95% CI:-0.72, -0.43), respectively. Allowing the effect of solid organ transplant recipients in outcomes of PWoH could result in ecological fallacy, as this group is substantially immunocompromised and clinically complex compared to the average PWoH. For example, immunosuppressants are administered to prevent organ rejection and promote recovery following solid organ transplantation. These immunosuppressants may lead to suboptimal vaccine responses. Additionally, the immunosuppression arising from having a severe chronic illness increases risk for infections in SOT recipients [47]. Therefore, we concluded that our data on PWoH would be a more appropriate comparator if the data on SOT was excluded from our meta-analysis comparing PWH to PWoH.

**Fig 5:**
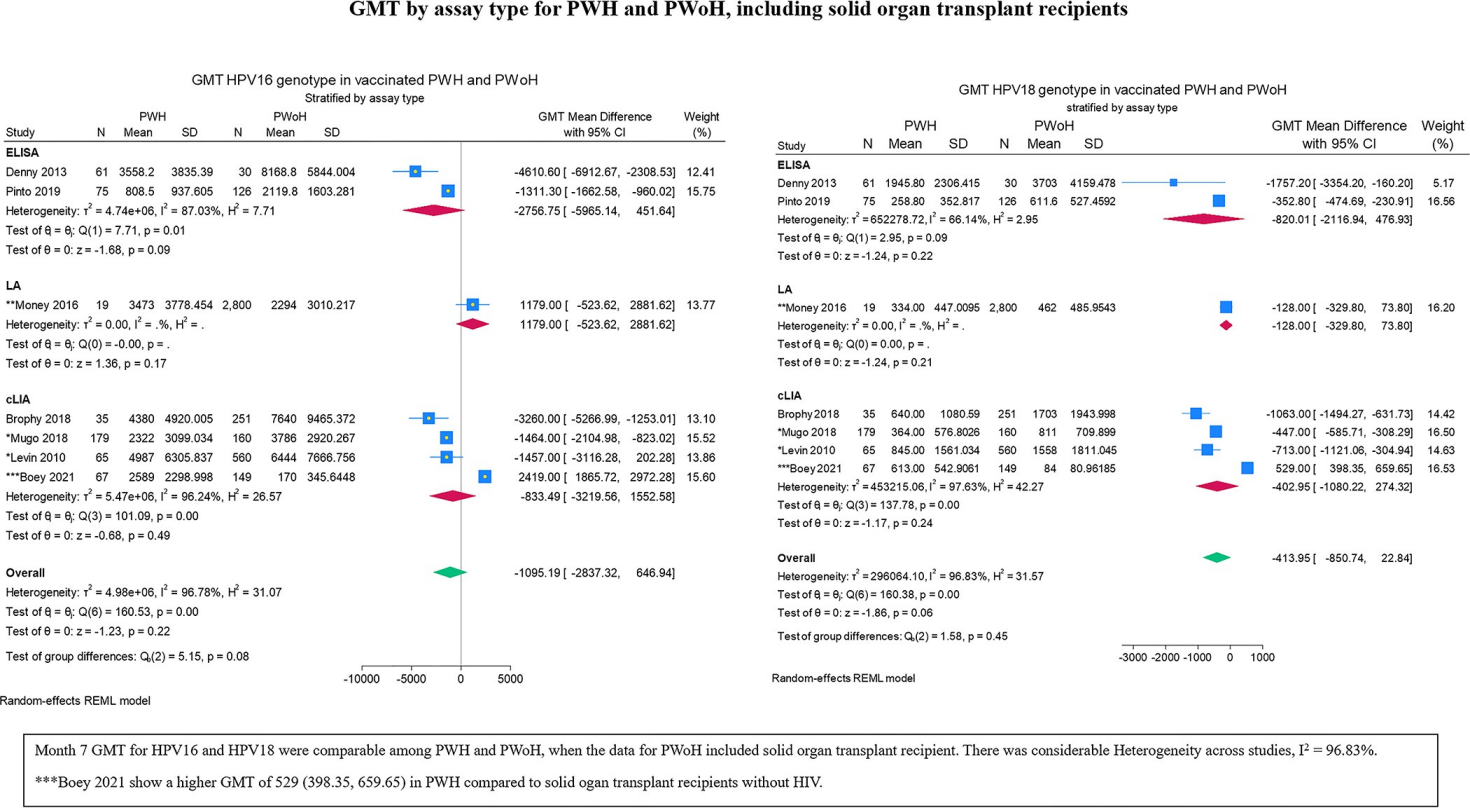
GMT by assay type for PWH and PWoH including SOT recipients.

**Fig 6:**
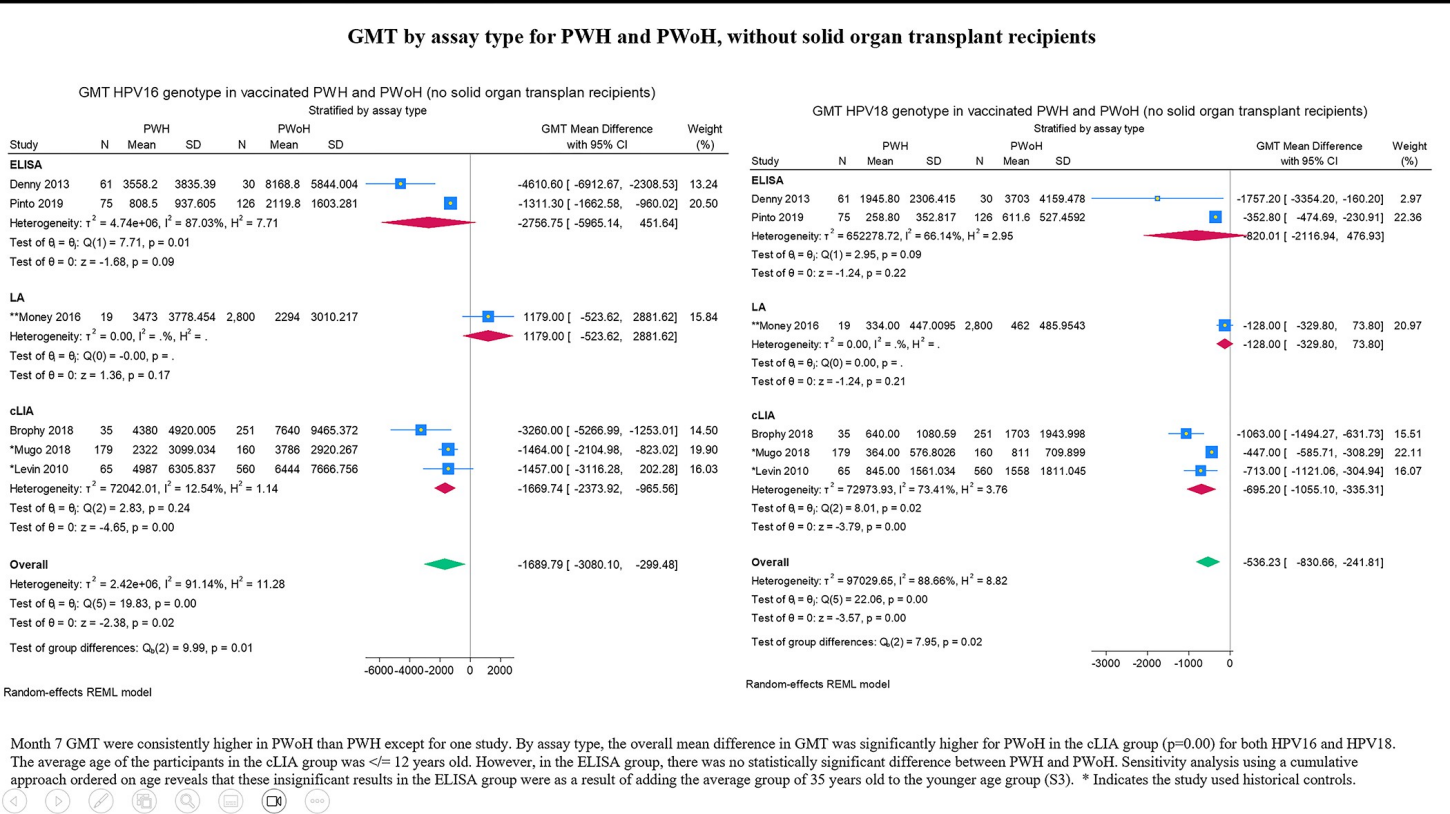
GMT by assay type for PWH and PWoH, solid organ transplant recipients not included.

**Fig 7:**
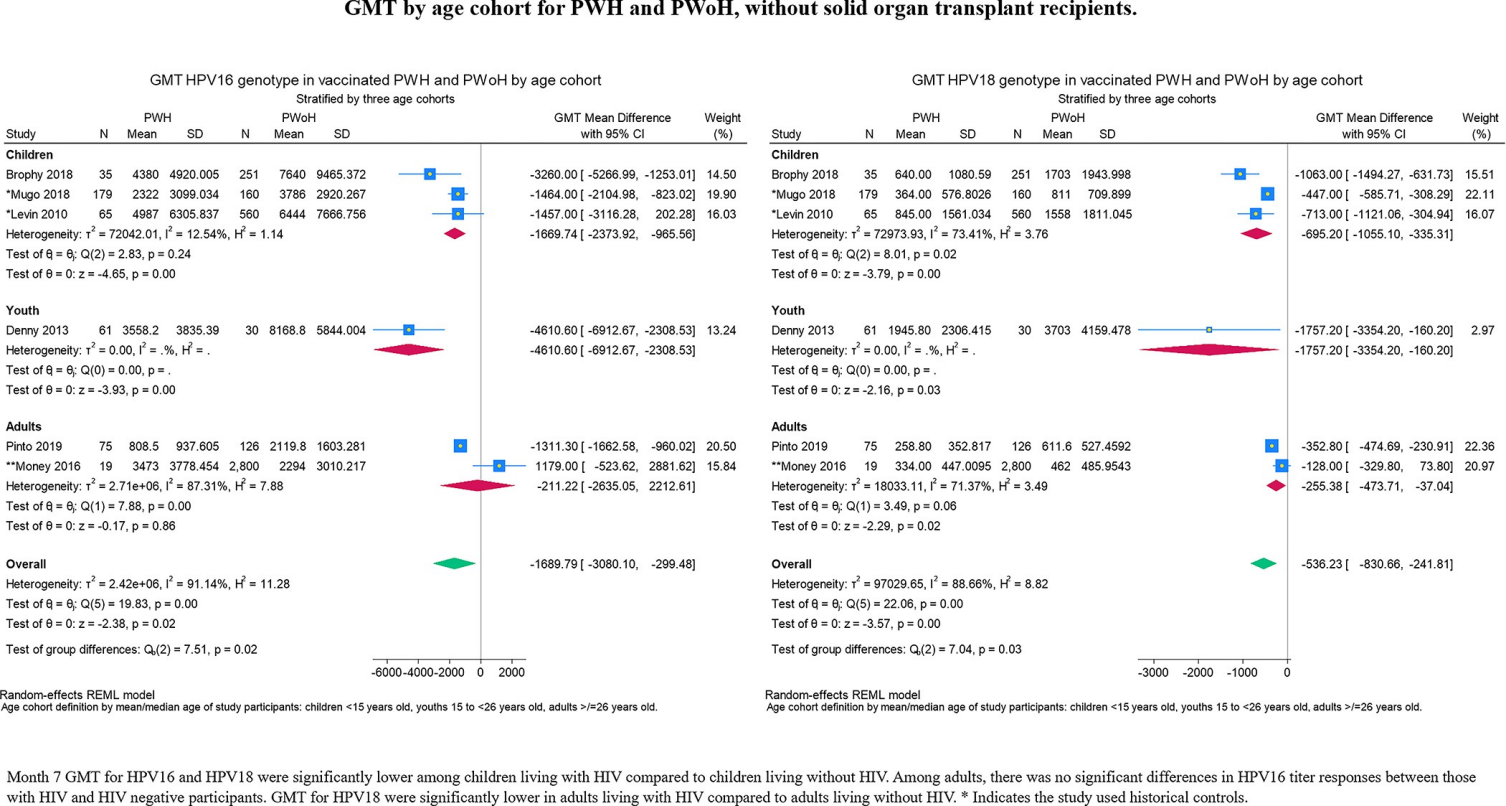
GMT by age cohort for PWH and PWoH.

Unstandardized mean differences in GMT are reported in forest plots in preference to Hedge’s g due to the potential clinical relevance of the former. GMT by assay type is presented in **Fig 5** which includes the study with SOT recipients. For subsequent forest plots, we do not account for the contribution of SOT recipients to the data for PWoH. Differences in mean GMT were generally lower in PWH. By assay type, the difference between PWH and PWoH was insignificant in the ELISA group, p = 0.09 and p = 0.22 for HPV16 and HPV18, respectively (**Fig 6**). Sensitivity analysis using a cumulative approach ordered on age reveals that these insignificant results in the ELISA group were a result of adding the average group of 35 years old to the younger age group (S1 Fig). Analyses stratified by age cohorts suggest that there is no significant difference in HPV16 response between adults living with HIV and adults living without HIV -211.22 (95% CI: -2635.05, 2212.61; **Fig 7**). Conversely, this difference was significant in the response to HPV18 across all age categories, despite lower GMT levels overall (**Fig 7**). Among children (<15 years old) and youth (15-to-26 years old), the differences in mean GMT for both HPV strains were significantly lower in PWH compared to PWoH. For differences in GMT response to HPV16 by HIV serostatus, p 0.00 for both children and youth. For differences in GMT response to HPV18 by HIV serostatus, p = 0.00 for children and p = 0.03 for youth.

**Fig 8:**
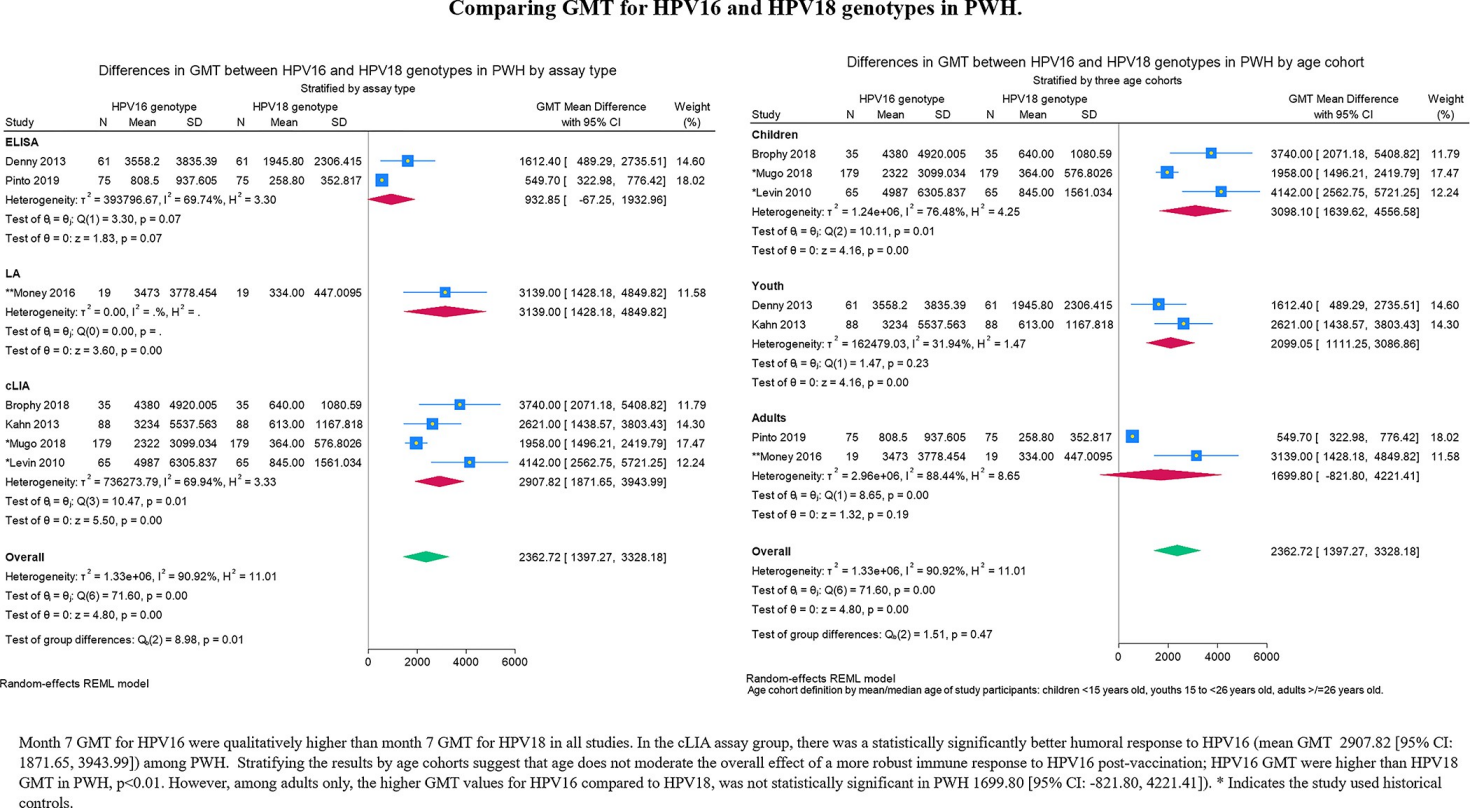
Difference in GMT for HPV16 and HPV18 in PWH.

The mean difference in GMT between PWH and PWoH for HPV18 was –536.23 (95% CI: -830.66, -241.81; **Fig 7**), which is approximately 22 times higher than the GMT cutoff point of seropositivity for HPV18. The mean difference of GMT in PWH was consistently higher for HPV16 than HPV18 (**Fig 8**).

#### Durability of immunogenic protection in PWH

Among PWH, factors associated with long-lasting seropositivity included undetectable HIV viral load, higher CD4+ T cell count, and sustained immunologic response after the third HPV vaccine dose. Cut-off points for undetectable HIV viral load were inconsistent across studies. HIV RNA viral load of <400 copies/mL was the most significant predictor of immunogenicity across studies. CD4+ T-cell counts ≥ 250 cells/mm3 were also significantly associated with higher antibody responses post-vaccination.

Antibody concentrations at week 28 (4 weeks after third dose; also referred to as month 7 in some studies) predicted persistent antibody responses at 96 weeks for all HPV genotypes *(p <* .*0001*) [46]. Across CD4+ T cell strata, there was a decline in seroconversion rates from week 28 to week 72 [49]. Women with a CD4+ T cell count of >350 cells/mm^3^ maintained higher seropositivity trends compared to women with a CD4+ T cell count of ≤200 cells/mm^3^ *p = 0*.*016*) [49].

*Trends in duration of protection after HPV vaccination*. In studies where duration of protection was assessed, GMT peaked at month seven, with a marked decrease observed at month twelve for all PWH. The 2vHPV vaccine induced a more durable immunologic response compared to 4vHPV, particularly for the HPV18 genotype [38,39]. Seropositivity for all vaccine types and HPV genotypes increased from baseline to month seven. After this, there was a decrease in seropositivity for HPV genotypes in all studies, with a plateau between month 18 and month 24. HPV vaccines induced a more persistent seropositivity to HPV16 than HPV18. However, 18 months after the third dose, a fourth dose of the 4vHPV vaccine administered to children with HIV resulted in seropositivity of 96% to HPV18, as opposed to seropositivity of 76% to HPV18 in children living with HIV who received three doses [46]. This suggests that booster doses of HPV vaccines in PWH may increase protection. However, a follow-up on the study revealed that HPV-specific B-cell and T-cell memory declined over time in children who received both three and four doses, with no significant differences between the vaccination groups [36].

#### HPV vaccine-induced cellular immunity in PWH

The role of HPV vaccine-induced cell-mediated immunity could inform a more comprehensive outlook on protective pathways of HPV vaccines in PWH. Cellular immune responses induced by vaccination of PWH were measured by two clinical trials across three publications [36]. Despite the fading of circulating antibodies over time, the evidence is suggestive of the persistence of B-cell and T-cell memory responses up to 5 years post-vaccination. Nevertheless, T-cell function declined over time, and this was negatively correlated with HIV plasma RNA load [36,43,46]. PWH with lower HIV viral load or higher CD4+ T cell counts at baseline developed better T-cell responses to HPV16 and HPV18. The positive correlation between T-cell responses and CD4+ T-cell counts were more significant in a sample with a higher percentage of participants on ART (96%), compared to a sample with less participants on ART (74%) [43]. Significant increases in T cell frequencies for cross-reactive antigens were also observed among recipients of both 2vHPV and 4vHPV vaccines, without indication of any of the vaccines demonstrating immunologic superiority [43].

#### Responses by sex

There was a trend towards a more robust immune response against oncogenic HPV strains to the 2vHPV vaccine among women. In an RCT consisting of women with a mean age of 19.8 (SD; 3.2), 2vHPV vaccine demonstrated immunologic superiority over 4vHPV vaccine against HPV16 and 18 strains [39]. Antibody titers were significantly higher in 2vHPV vaccine recipients compared to 4vHPV vaccine recipients (*p < 0*.*0001*) [39]. Meanwhile, there were no observable differences in antibody titers between 2vHPV and 4vHPV vaccines in men [44].

No differences in antibody titers were observed between men and women who received the 4vHPV vaccine [44]. Sex-stratified analyses suggested immunologic superiority of 2vHPV in women compared to men, with a *3*.*16-fold* higher anti-HPV16 antibody titers one month after the third dose in women than men [44]. Cross-reactive antibodies were higher in females than males who received 2vHPV vaccine [64]. GMT for HPV 31/33 were higher for women compared to men at months seven and twelve [45].

### iii. Overview of HPV vaccine efficacy in PWH

Efficacy of HPV vaccines in PWH was evaluated across seven trials and eleven publications [21,40,41,44,45,49,54,55,57,59,61]. One of the publications did not report on post-vaccination efficacy endpoints but did estimate the risk factors associated with high-risk HPV genotypes at baseline [40]. Two publications measured either cross reactive HPV or non-4vHPV vaccine oncogenic HPV [54,56]. Eight publications reported HPV vaccine efficacy clinical endpoints of post-vaccination HPV infections, CIN, and or SIL outcomes [21,41,49,54,57,59,61,65]. Two of these reported on CIN outcomes and two measured HSIL. Studies of incident and persistent anal, cervical, and oral HPV 16/18 genotypes are presented in **Table 2**.

**Table 2:** Overview of studies of HPV vaccine efficacy for HPV16 & HPV18 infections.

Studies measuring incident and persistent HPV16/18 infections					Site
Author	Incident HPV infection		Persistent HPV infection		
	16	18	16	18	
**Cespedes 2018 [49]**	☑	☑	☑	☑	**Anal**
	☑	☑	☑	☑	**Cervical**
**McClymont 2019 [54]**	x	x	x	x	**Anal**
	x	x	☑	☑	**Cervical**
**Hidalgo-Tenorio 2021 [41]**	☑	☑	x	x	**Anal**
	x	x	x	x	**Cervical**
**Palefsky 2021 [59]**	☑	☑	☑	☑	**Anal**
	x	x	x	x	**Cervical**
**Toft & Storgaard 2014 [44]**	☑	☑	☑	☑	**Anal**
	☑	☑	☑	☑	**Cervical**
**Wilkin 2010 [61]**	☑	☑	☑	☑	**Anal**
	x	x	x	x	**Cervical**
**Wilkin 2018 [21]**	☑	☑	☑	☑	**Anal**
	x	x	x	x	**Cervical**
	☑	☑	☑	☑	**Oral**

☑ HPV testing performed

x = HPV testing not performed.

### iv. Efficacy of HPV vaccines in PWH

#### HPV infections

Both cervical and anal incident HPV infections were uncommon in participants who were HPV DNA negative at baseline, irrespective of type of vaccine received [45,49,65]. Among males living with HIV, 5% developed incident HPV18 infection after the 2vHPV vaccine, and 3% developed incident HPV18 infection after the 4vHPV vaccine [65]. Additionally, 2% developed incident HPV16 to 2vHPV vaccine, and 3% developed incident HPV16 infection to 4vHPV vaccine. Meanwhile, none of the WLWH in this study developed any incident infections [65].

Persistent HPV infections across studies were defined as 2 or more consecutive positive results of the same HPV genotype > = 6 months apart [21]. Though a negligible occurrence, anal HPV DNA was more prevalent than cervical HPV DNA in women who were HPV DNA negative at baseline. At week 52, one out of 286 had persistent cervical HPV16 infection while three out of 281 women had persistent anal HPV18 infections [49].

One study measured oral HPV infections with a suggested vaccine efficacy of 88% for preventing oral infections [21]. This study also reported an HPV vaccine efficacy of 22% for prevention of anal HPV infections. It is noteworthy that the study population had high levels of prevalent anal HPV infections at baseline and the trial was stopped early. Similarly, another study of men living with HIV reported a high prevalence of anal HSIL at baseline, resulting in exclusion of 34% of participants that were screened for this study [59].

#### Squamous intraepithelial lesions

Two studies measured vaccine efficacy on high-grade squamous intraepithelial lesions. Palefsky and colleagues found that among men living with HIV, there were no incident vaccine-type anal squamous intraepithelial lesions [59]. This was irrespective of a 47% prevalence of HPV16 in those enrolled in the study. On the contrary, Hidalgo-Tenorio and colleagues reported null results of rates of HSIL between vaccinated and placebo arms of men living with HIV [41]. It is also noteworthy that 30% of participants in this study had a history of AIDS.

#### Neoplasia outcomes

A trend towards conflicting results was also observed in the reporting of neoplasia outcomes. One study among the non-RCTs measured high-grade AIN (HGAIN) [61]. Progression from no AIN to HGAIN was observed in 10% of participants, and progression of low-grade AIN to HGAIN was 22% [61]. This study was not specifically designed for efficacy and participants had a median age of 44 years old, with 14% having a history of AIDS-defining illness.

Of the two efficacy studies that measured cervical intraepithelial neoplasia (CIN) as an outcome [54,57], one evaluated grade 2+ (CIN 2+) [54]. No cases of CIN 2+ were observed in women with baseline normal cervical cytology. This was the first study to report CIN2+ outcomes in women and the median follow-up time was 2 years [54]. Participants had a median age of 39 years old. Eligibility criteria included excluding participants whose health was considered exclusionary by a site investigator [54]. The implications of this exclusionary criterion remained unclear, even though it might have been beneficial to the efficacy results. The extent to which eligibility criteria in clinical trials of HPV vaccines in PWH influence the conflicting results in efficacy outcomes needs further inquiry.

## IV. Discussion

This review indicates that post-vaccination titers are lower for PWH compared to PWoH and further investigates factors influencing HPV vaccine efficacy in PWH. Evidence on the efficacy of HPV vaccines has been sparse and inconclusive [24,66–68]. Nevertheless, our review of both randomized and non-randomized clinical trials provided insights into efficacy results not previously discussed in other reviews of RCTs. Our in-depth exploration of HPV vaccine efficacy in PWH sheds light on variables not typically considered in estimating HPV vaccine failure.

### 1. Factors associated with HPV vaccine failure in PWH

Vaccine failure in our review was related to efficacy endpoints and defined as the development of incident or persistent vaccine-specific oncogenic HPV infections or intraepithelial neoplasia after three doses of HPV vaccines. Detection of type-specific HPV infections post-vaccination was uncommon in persons who were HPV DNA negative at baseline.

#### Risk factors associated with HPV vaccine failure

Risk factors resulting in the development of incident or persistent infections included: failure to seroconvert, baseline CD4+ T cell count <500 cells/mm3, early age of sexual debut, HIV viral load ≥ 200 copies/mL, and being male (increased the likelihood of both incident and persistent anogenital HPV infections compared to being female) [40,45,49,54,65]. Anal incident or persistent HPV was more likely to occur than cervical infections [49,65]. Persistence was more likely with HPV18 [54]. Whether this increased persistence in HPV18 is related to the lower overall GMT for HPV18 we found in our quantitative synthesis remains to be researched. Additionally, a history of AIDS or AIDS-defining illness could be suggestive of being at risk of HPV vaccine failure as observed in the lower efficacy of HPV vaccines in studies with participants meeting this criterion at baseline [41,61].

Some non-infectious and infectious co-factors for HPV cancers are yet to be explored by clinical trials of HPV vaccines in PWH. Significant non-infectious co-factors include smoking, parity, nutrition, hormones, and hormonal contraceptive use [69]. Potentially infectious co-factors that are worth exploring in PWH include herpes viruses and *Chlamydia trachomatis*.

#### Protective factors

Significant protective factors against post-vaccination HPV infections included condom use, HPV DNA negative at baseline, and not having vaccine-type SIL [41,54,59]. None of the participants with baseline normal cytology developed CIN 2+ at two years of follow-up [54]. Consequently, the latter results are indicative of the protective benefits of a negative baseline Papanicolaou test on HPV vaccine efficacy in adults living with HIV [54]. Age remains a major confounder in HPV vaccine efficacy due to the high probability of existing infections, yet catch-up vaccination of PWH through 26 years old may be beneficial [59].

In addition to informing our understanding of drivers of HPV vaccine efficacy in PWH, the results of our review have research, practice, and policy implications. Our recommendations for practice focuse on the U.S. because of authors’ depth of knowledge and practice experiences are within the U.S.

### 2. Practice and policy implications

The data from this review begs the question if vaccination should be delayed in specific cohorts until viral suppression is achieved. The tradeoff between missing the opportunity to vaccinate if vaccination is delayed and attaining viral suppression is a vital consideration. Delaying vaccination until after CD4+ rebound increases the risk of exposure to oncogenic HPV strains and potentially reduces the clinical and economic benefit of vaccination. Where loss to follow up concerns are present, any opportunity to vaccinate should be prioritized. In such instances, clinicians should encourage vaccine administration during any contact with a healthcare setting, such as during hospitalization for acute illness, or in non-acute care settings.

Clinicians may consider using viral suppression as a clinical predictor of vaccine efficacy rather than CD4+ count as previously recommended with other vaccine series [70]. Given the shortened time to viral suppression with the use of integrase inhibitors, a short delay in vaccination for a more robust vaccine response may be warranted. This short delay could improve outcomes of HPV vaccines in PWH, by boosting clearance of HPV infections then preventing progression to neoplasia. [71].

In the U.S. for example, where HPV vaccines are available for adults, personalized clinical decision-making regarding optimal vaccination of adults living with HIV is a necessity. The 9vHPV vaccine is FDA-approved for adults up to age 45 [72]. The CDC recommends shared decision-making regarding HPV vaccination for persons who are 27-to-45 years old in the U.S. [73]. However, there are no clear guidelines to assist providers with informed shared decision-making, especially at the point of care. Providers may wish to share data from this review about viral suppression/CD4 with their patients. The proposed risk and protective factors of vaccine failure suggested by this review could be particularly beneficial to clinicians caring for PWH.

Further discussion may be warranted regarding the increased rates of breakthrough infections among men living with HIV compared to women. This is the ideal group to engage in meaningful patient-provider discussion regarding HPV infection. Adults in the 27–45 age group have more developed executive functioning than adolescents and can theoretically engage in meaningful discussions about their health. Given the current culture surrounding vaccination, honest discussions about costs vs benefits and efficacy may help to improve patient-provider trust.

New HIV infections in the U.S. are highest among the 25-to-44-year-old age cohort straddling this HPV clinical vaccination guideline [74]. HPV vaccines were first recommended for adolescent girls in 2006 and adolescent boys in 2011. As a result, many individuals in the 25–44 cohort likely missed HPV vaccination as adolescents or young adults. Special attention should be paid to this age group as they are at risk for HIV and HPV infection. HIV care providers and primary care providers servicing PWH must be aware of the possible vaccination gap and their resulting vulnerability to HPV infection and its sequelae.

There is no significant risk of vaccination in women in the 27–45 age cohort that would preclude this group from vaccination. Though most women have been exposed to HPV by adulthood, it is unlikely that they have been exposed to all the strains covered by the 9vHPV vaccine leaving them vulnerable to new and persistent preventable infections. Guidelines implemented by the Affordable Healthcare Act mandate that private insurance companies reimburse HPV vaccines chosen via shared decision-making eliminating one possible barrier to vaccination [75]. Despite improving access to HPV vaccination for women 27–45 in the United States, access may be limited or restricted in lower-resource countries with higher rates of HIV prevalence. The projected incidence in oropharyngeal cancer through 2045 indicates a shift in burden to an older population of adults [76]. The findings from this study and the known risks of co-infection with HIV and HPV underscores the importance of increasing vaccination rates for eligible PWH globally. Coupled with the plateau of titer levels, these findings further raise the question if HPV vaccine booster doses may be beneficial.

### 3. Research implications

HPV vaccine efficacy in PWH remains inadequately investigated, worse in some regions than others. Ethically, it is now questionable to implement new placebo-controlled trials in evaluating efficacy of HPV vaccines in PWH. This reason was also stated as a rationale in one of the more recent publications included in this review [59]. Comparative efficacy trials, pragmatic trials, and prospective longitudinal trials would be an equitable way forward. HPV vaccine effectiveness studies would also provide valuable insights into practical and sustainable “real life” implications of vaccination. Advances in artificial intelligence (AI) and precision medicine provide novel opportunities for innovative approaches to assessing HPV vaccine failure as an alternative to RCTs. This is particularly crucial as it is obvious from existing literature that RCTs may be an impractical approach to reliably evaluate HPV vaccine efficacy. The unique nature of HPV-associated cancers to have a long duration from infection to malignancy hinders cost-effective implementation of RCTs that accomplish this goal.

#### AI and precision medicine in HPV vaccine clinical trials of PWH

Data-driven AI tools have mostly been used to facilitate the execution of clinical trials such as using elecronic medical records data to suggest suitable matches between patients and research studies. In a world of unlimited data, AI presents a unique potential for greater statistical power without the intensive manual expectation of traditional RCTs. It is suggested that AI may permit the use of a synthetic control arm due to the ability to predict the natural history of each participant [77]. Therefore, the ethical concerns from the use of placebo vaccines in traditional RCTs designs may be averted using well-designed AI tools. Current challenges to efficient adaptation of AI and precision medicine tools are limited by the quantity and quality of existing RCTs.

Effective implementation of precision medicine tools requires the identification of precise observable variables that predict outcomes after HPV vaccines are administered to PWH. Precision medicine accounts for variation of genes, environment, and lifestyle [78]. Therefore, the mapping of the human genome has presented inconceivable potentials in characterizing associations between the biological traits of PWH and their immune responses to HPV vaccines. Extra attention should also be paid to patient-reported data, including establishing correlations between patient-reported adverse events with immunogenicity and efficacy of HPV vaccines. Should researchers choose to explore these relationships, existing data from HPV vaccine clinical trials present a cost-effective approach. There are also opportunities to learn a lot from the outlier effects that are so often disregarded or how psychosocial inclusion criterion predict outcomes within studies of HPV vaccine responses in PWH. Future studies could also adapt interventions according to individual responses following each dose of HPV vaccine administration. While this may not be cost-effective, it offers a more equitable and forward-thinking option for vaccine clinical trials in PWH.

#### The role of eligibility criteria

Though the efficacy results of HPV vaccine trials have been inconsistent, our in-depth review and analysis suggests the need for considerations of the eligibility criteria used across clinical trials to inform policy recommendations and outcome reporting standards. While this was outside the scope of our review, it is worth questioning if the eligibility criteria significantly influence these variations in HPV vaccine efficacy in PWH or if the overall results from all studies provide globally representative efficacy data that are more generalizable.

#### Preventing incident infections vs disability in adults

Finally, given their prophylactic use, HPV vaccines do not protect against infections that are already present at time of vaccination. With relatively high baseline prevalence of oncogenic HPV strains prior to vaccination, the question if adults living with HIV should be vaccinated is mind boggling. A reality that necessitates deliberate interrogation of not only the measurable costs but also the psychosocial implications. More data is needed to wholly inform these decisions such as additional research on the potential benefits of preventing vaccine-type HPV strains that are not present at baseline. Another consideration would be if post-vaccination incident infections progress to clinically disabling conditions. If HPV vaccines prevent incident infections from progressing to malignancy, then discussions and comprehensive analyses of longer-term benefits may be warranted.

## V. Limitations

Only publications available in English were reviewed which could have introduced some degree of selection bias. This might explain the absence of clinical trials from Eastern Europe and Central Asia, which are the regions with the most rapid rise in HIV cases since 2010 [79]. Meanwhile, in sub-Saharan Africa 3,100 adolescent girls and young women (aged 15 to 24 years) become infected with HIV every week [79]. Yet, only South Africa and Kenya were included in the studies of HPV vaccine trials in PWH. While the limited representation of these vulnerable regions could have resulted from our inclusion of publications available in the English language only, that is an unlikely reason. English is an official language in twenty-six countries in sub-Saharan Africa [80].

One limitation of the meta-analysis was the relatively small sample sizes of the clinical trials. The sample size of PWH ranged from 19 to 175 in studies that reported on HPV vaccine-type specific genotype GMT at month 7 (Fig 5). While impactful policy decisions have been made from the results of these clinical trials, there may be some concerns with generalizability. The sample sizes of these trials could also be a reason efficacy results continue to remain inconclusive.

Furthermore, the absence of standardized reporting measures for HPV vaccine clinical trials limits the number of studies in any quantitative synthesis of HPV vaccine outcomes in PWH. This is suggestive of the need for evidence-informed standardized reporting measures in HPV vaccine clinical trials to assist in streamlining evidence synthesis and promoting translation of evidence to clinical practice.

## VI. Conclusions

The applicability of the existing evidence of HPV vaccine efficacy on meaningful clinical outcomes remains questionable. A need exists for evidence-informed standardized reporting measures in HPV vaccine clinical trials of PWH. Streamlining evidence synthesis will promote translation of evidence to clinical practice. Though the HPV vaccines are highly immunogenic, titer levels wane over time. Persistence of oncogenic HPV strains that can be prevented by all available HPV vaccines (HPV16/18) is higher with HPV18 genotype than HPV16 genotype. This correlates with much lower HPV18 GMT over time in those vaccinated. Consideration of booster shots in the long run may be worth exploring. There may be limited merit to vaccination at the population-level for adults with HIV. However, for those who could benefit from HPV vaccines, the advantages warrant vaccination to promote equitable access and prevent illness and disability from HPV vaccine-preventable ailments. Longer follow-up time will better inform vaccination schedules in adults to determine if type-specific HPV infections acquired post-vaccination progress to malignancy. Vaccination of adolescents and young adults remain the most efficacious and effective option for PWH.

## Supporting information

S1 Checklist PRISMA 2020 checklist. (DOCX)

S1 Fig Cumulative meta-analysis. (TIF)

S1 Table Search strategy. (XLSX)

S2 Table Summary of immunogenicity information. (XLSX)

S3 Table 774 records; including those excluded from our analyses/review. (XLSX)

## Abbreviations

Abbreviation: Meaning
AIN: Anal Intraepithelial Neoplasia
ART: Antiretroviral Therapy
CI: Confidence Interval
CIN: Cervical Intraepithelial Neoplasia
cLIA: Competitive Luminex Immunoassay
ELISA: Enzyme-linked immunosorbent assay
FDA: U.S. Food and Drug Administration
GMT: Geometric Mean Titers
HIV: Human Immunodeficiency Virus
HPV: Human Papillomavirus
mMU/ml: milli-Merck Units per milliliter
Non-RCT: Nonrandomized clinical trials or open label trials
PWH: People Living with HIV
PWoH: People Living without HIV
RCT: Randomized controlled trials
SIL: Squamous intraepithelial lesions
SOT: Solid Organ Transplant
UNAIDS: The Joint United Nations Programme on HIV/AIDS
U.S.A./U.S.: United States of America
WHO: World Health Organization
WLWH: Women Living with HIV
WLWoH: Women Living without HIV
