# Supplementary figures and images for: Understanding human papillomavirus vaccine response and efficacy in people living with HIV: A systematic mixed studies review and meta-analysis
Source: PLOS Glob Public Health. 2024 Dec 20;4(12):e0003931. doi: 10.1371/journal.pgph.0003931 (PMC11661617; doi:10.1371/journal.pgph.0003931)

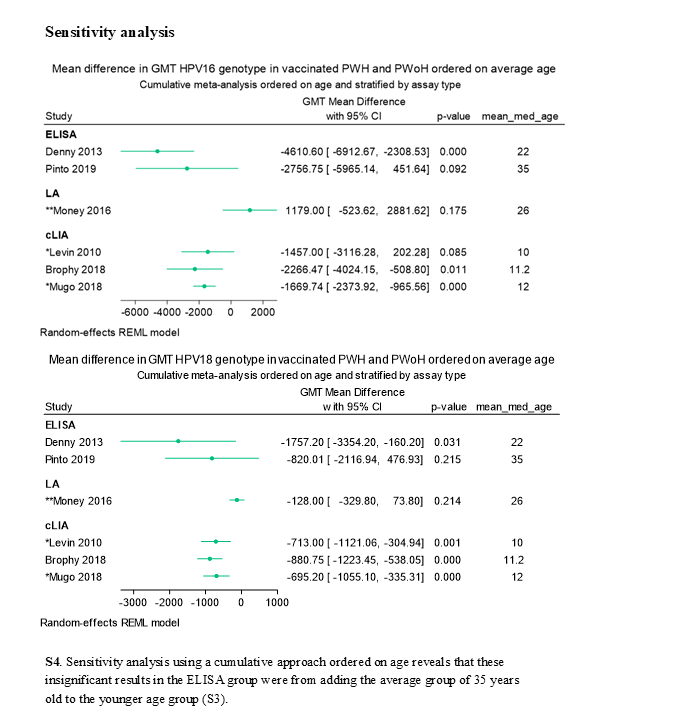

Supplement: S1 Fig — (TIF) [file pgph.0003931.s002.tif]
